# Sulfonium Acids Loaded onto an Unusual Thiotemplate Assembly Line Construct the Cyclopropanol Warhead of a *Burkholderia* Virulence Factor

**DOI:** 10.1002/anie.202003958

**Published:** 2020-05-29

**Authors:** Felix Trottmann, Keishi Ishida, Jakob Franke, Aleksa Stanišić, Mie Ishida‐Ito, Hajo Kries, Georg Pohnert, Christian Hertweck

**Affiliations:** ^1^ Department of Biomolecular Chemistry Leibniz Institute for Natural Product Research and Infection Biology (HKI) Beutenbergstrasse 11a 07745 Jena Germany; ^2^ Junior Research Group Biosynthetic Design of Natural Products Leibniz Institute for Natural Product Research and Infection Biology (HKI) Beutenbergstrasse 11a 07745 Jena Germany; ^3^ Institute of Botany Leibniz University Hannover 30419 Hannover Germany; ^4^ Institute for Inorganic and Analytical Chemistry Friedrich Schiller University Jena 07743 Jena Germany; ^5^ Natural Product Chemistry Faculty of Biological Sciences Friedrich Schiller University Jena 07743 Jena Germany

**Keywords:** Biosynthesis, DMSP, Mass spectrometry, NRPS, Virulence factors

## Abstract

Pathogenic bacteria of the *Burkholderia pseudomallei* group cause severe infectious diseases such as glanders and melioidosis. Malleicyprols were identified as important bacterial virulence factors, yet the biosynthetic origin of their cyclopropanol warhead has remained enigmatic. By a combination of mutational analysis and metabolomics we found that sulfonium acids, dimethylsulfoniumpropionate (DMSP) and gonyol, known as osmolytes and as crucial components in the global organosulfur cycle, are key intermediates en route to the cyclopropanol unit. Functional genetics and in vitro analyses uncover a specialized pathway to DMSP involving a rare prokaryotic SET‐domain methyltransferase for a cryptic methylation, and show that DMSP is loaded onto the NRPS‐PKS hybrid assembly line by an adenylation domain dedicated to zwitterionic starter units. Then, the megasynthase transforms DMSP into gonyol, as demonstrated by heterologous pathway reconstitution in *E. coli*.

## Graphical abstract

**Sulfur cycle meets pathogen**. Sulfonium acids known from global sulfur cycles were elucidated as the biosynthetic origin of the cyclopropanol warhead of malleicyprols, virulence factors of human‐pathogenic bacteria. The pathway involves a specialized *S*‐methyl transferase to form DMSP, which is activated by a zwitterion‐specific adenylation domain and transformed into the key intermediate gonyol.

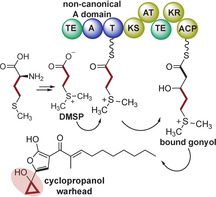

*Burkholderia pseudomallei* group pathogens cause lethal infections that are difficult to treat.^1^ The best‐known members of this pathogen complex are *Burkholderia mallei*, which causes the zoonotic disease glanders, and *B. pseudomallei*, the causative agent of melioidosis. The low infective dose needed and the possibility of infections through inhalation have led to the classification of *B. mallei* and *B. pseudomallei* as biological warfare agents^2^ and a threat to global health.^3^ Since infections by these notorious pathogens are difficult to treat,^4^ novel therapeutic approaches such as anti‐virulence strategies are needed. As prerequisite to disarming pathogens, it is essential to understand their virulence factors and the biosynthetic pathways involved.^5^ For *B. pseudomallei* and related pathogens such as the less virulent model organism *B. thailandensis*, various macromolecular^6^ and low‐molecular‐weight virulence factors^3^, ^7^ have been identified. Notably, all pathogens of the *B. pseudomallei* complex share a gene locus coding for an unusual modular thiotemplate assembly line with components of modular non‐ribosomal peptide synthetases (NRPS) and polyketide synthases (PKS). Gene inactivation experiments unequivocally linked this gene cluster, named *bur*, to pathogenicity in nematode^8^ and mouse models.^9^ However, the first metabolite associated to this pathway—burkholderic acid^10^ *syn*. malleilactone^8^ (**1**)—did not exhibit any activity explaining the phenotypes observed in the infection models.

Recently, we found **1** to be the inactivated form of the true virulence factor, a highly reactive, cyclopropanol‐substituted congener named bis‐malleicyprol (**2 a**, Figure 1 A) formed from the monomer malleicyprol (**2 b**).^11^ Nematode and toxicity assays showed dramatically increased activity of **2 a** compared to **1**, implicating the cyclopropanol warhead in virulence.^11^ Thus, understanding its biosynthesis would set the basis for antivirulence strategies. According to stable‐isotope labeling experiments and gene knockouts, the NRPS‐PKS hybrid enzyme BurA assembles the cyclopropanol‐containing fragment of **2 b**, followed by dimerization to **2 a**, which opens to form the inactive propanone‐substituted unit of **1**, from a yet unknown methionine (**3**)‐derived C3 building block and malonyl‐CoA (Figure 1 B).^10^ Yet, structures and biotransformations of the precursors loaded onto BurA have remained a riddle. Here we decipher the biogenetic origin of the rare cyclopropanol warhead of malleicyprol and show that a set of zwitterionic sulfonium acids initiates biosynthesis that play key roles in global sulfur cycling.

**Figure 1:**
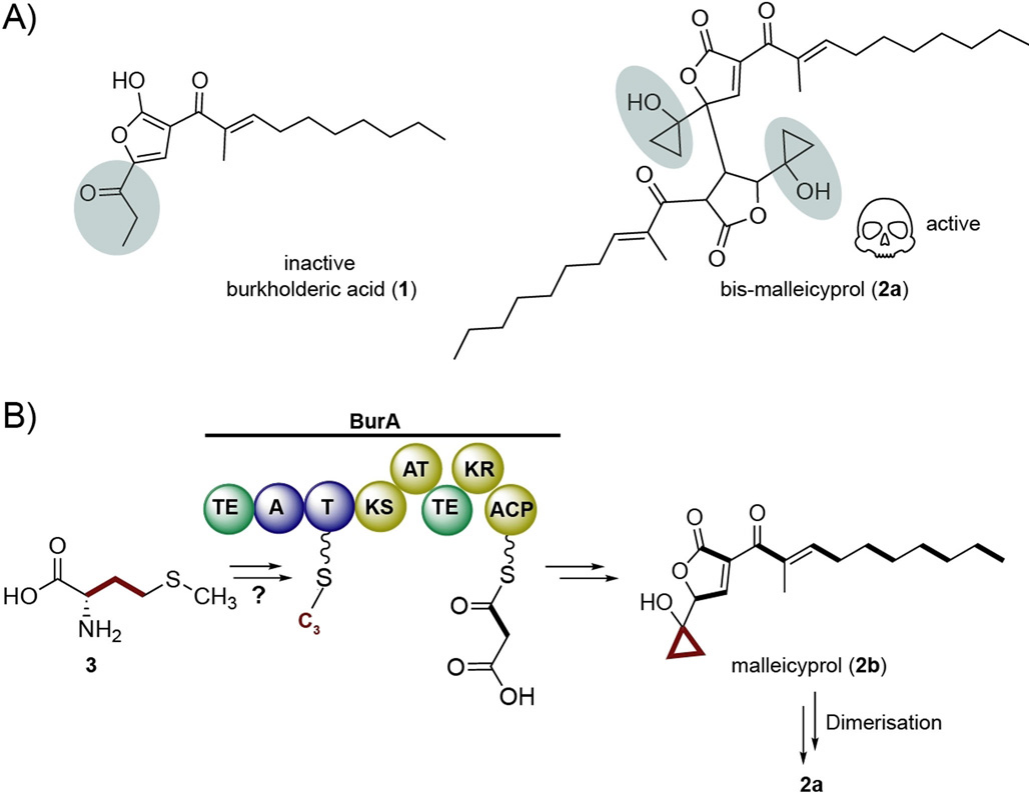
A) Structures of burkholderic acid (**1**) and bis‐malleicyprol (**2 a**) featuring a cyclopropanol warhead. B) Isotope‐labeling studies^10^ suggest acetate and methionine (**3**) as precursors to malleicyprol (**2 b**), the monomer^11^ of **2 a**.

To elucidate the biogenetic origin of the malleicyprol warhead we first focused on the NRPS‐PKS hybrid enzyme BurA and its unknown starter unit. Specifically, we tested for accumulation of intermediates in a mutant of the malleicyprol overexpressing strain *B. thailandensis Pbur* lacking a functional copy of *burA* (*B. thailandensis Pbur*Δ*burA* 10). Candidates for the sulfur‐containing pathway intermediates, however, could not be detected by routine HR‐LCMS‐based metabolic profiling. Only a comparative metabolomics analysis (*Pbur* vs. *Pbur*Δ*burA*) searching for highly polar metabolites revealed the elusive methionine‐derived starter unit. Compound **4** with *m*/*z* 135 accumulates in *B. thailandensis Pbur*Δ*burA* cells (Figure 2 A). Based on its exact mass (*m*/*z* 135.0474; [*M*+H]^+^) we deduced the molecular formula (C_5_H_11_O_2_S) of **4**. By comparison with authentic reference compounds we identified **4** as the zwitterionic compound dimethylsulfoniopropionate (DMSP; *m*/*z* 135.0474; [*M*+H]^+^; Supporting Information, Figure S1). This finding is intriguing since DMSP plays a pivotal role in the marine organosulfur cycle, serving as osmolyte for marine algae and as abundant carbon and sulfur source for bacteria.^12^ It is the precursor of the climate‐relevant gas dimethylsulfide that is emitted at remarkably high amounts of >10^7^ tons per year into the atmosphere.^13^ Despite its wide distribution, DMSP has thus far not been implicated as a building block in natural product biosynthesis.

**Figure 2:**
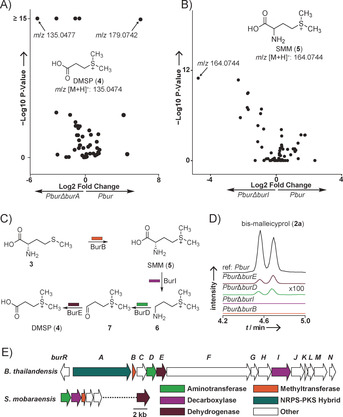
Identification of sulfonium intermediates in malleicyprol biosynthesis. A) Volcano plot analysis comparing pellet extracts of *B. thailandensis* variants *Pbur* and *PburΔburA*. B) Volcano plot analysis comparing supernatant extracts of *B. thailandensis* variants *Pbur* and *PburΔburI* (Figure S2). C) Proposed DMSP biosynthesis in *B. thailandensis*. D) Production of two bis‐malleicyprol (**2 a**, *m*/*z* 611.3589) diastereomers in gene inactivation mutants of *B. thailandensis Pbur* monitored by UHPLC‐MS (EIC in negative ion mode). E) Genomic alignment of the DMSP assembly line from *S. mobaraensis* to the *bur* biosynthetic gene cluster.

To confirm DMSP as a precursor of malleicyprols, we performed stable‐isotope labeling experiments by chemically complementing suitable block mutants. Therefore, we required insight into the molecular basis of DMSP formation in *B. thailandensis*. By analogy to one of the established bacterial DMSP biosynthesis pathways^12^ (see Ref. 14 for alternative routes to DMSP) methionine would undergo *S*‐methylation to form *S*‐methylmethionine (**5**), decarboxylation, transamination and oxidation (Figure 2 C). In silico analysis of the *bur* gene locus revealed candidate genes for a methyltransferase (BurB), a decarboxylase (BurI), a transaminase (BurD), and a dehydrogenase (BurE) (Figure 2 E). Comparison of the deduced protein sequences to the recently published bacterial DMSP biosynthetic machinery in *Streptomyces mobaraensis* 14b showed BurD and BurE to be almost identical with their orthologues (96 % and 98 %), whereas BurB and BurI are only distantly related to their *S. mobaraensis* counterparts (35 % and 48 %).

To disrupt malleicyprol production, we individually inactivated each of the four putative DMSP biosynthesis genes in *B. thailandensis Pbur* by homologous crossover and replacement with a resistance cassette. The Δ*burD* and Δ*burE* mutants are still capable of producing the malleicyprol complex, albeit in reduced amounts (Figure 2 D). Possibly, unspecific housekeeping enzymes take over transamination of sulfonium amine **6** and oxidation of the instable aldehyde **7**.14b In contrast, the two main malleicyprol diastereomers are absent in the Δ*burB* (methyltransferase) and Δ*burI* (decarboxylase) mutants (Figure 2 D).

Closer inspection of culture extracts from *Pbur*Δ*burI* revealed enrichment of metabolite **5** (*m*/*z* 164.0744 in positive ion mode) that is identical to the unusual charged amino acid *S*‐methylmethionine **5** (SMM; Figure 1 D and Figure S3). Since **5** cannot be detected in the mutant lacking *burB*, its gene product BurB must act in the biosynthetic pathway upstream of DMSP. BurB's role in DMSP formation is remarkable as it belongs to class V of the methyltransferase superfamilies containing a SET‐domain.^15^ SET‐domain‐containing methyltransferases are well studied in eukaryotes and regulate gene expression through histone lysine methylation.^15^ Yet, little is known about prokaryotic SET‐domain methyltransferases, and natural product modifying methyltransferases have exclusively been found in class I (Rossmann‐like fold) or class III (tetrapyrrole methylase).^16^ To corroborate the function of BurB, we cloned and overexpressed *burB* in *Escherichia coli* and purified the His6‐tagged protein via Ni‐affinity. Incubation of purified BurB with l‐methionine (**3**) and *S*‐adenosylmethionine (SAM) generated SMM (Figure 3 A), which was detected after derivatization with **8** to compound **9** (Figure 3 A). Thus, BurB represents a novel *C‐S* bond forming enzyme in secondary metabolism.^17^

**Figure 3:**
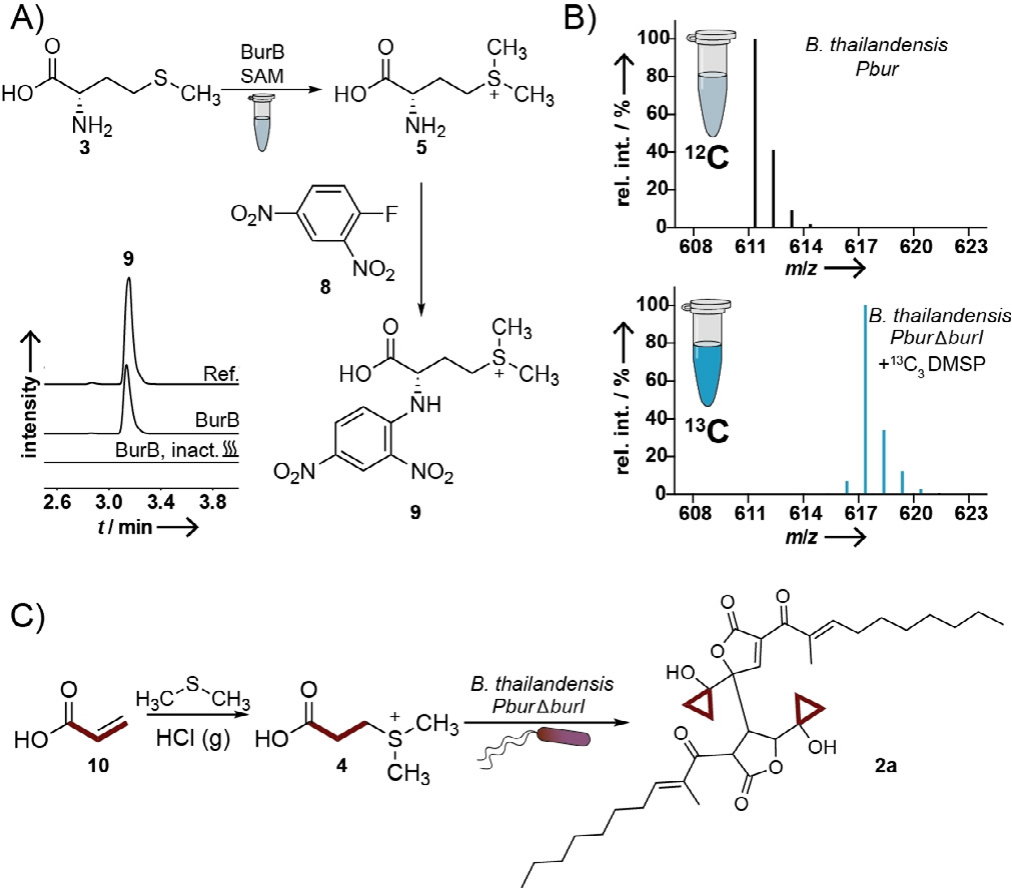
DMSP is a precursor of the cyclopropanol warhead of malleicyprol. A) Transformation of methionine (**3**) to *S*‐methylmethionine (**5**; SMM) by BurB and derivatization with **8**; HR‐LCMS detection of derivatized SMM; EIC *m*/*z* 330.0754 in positive ion mode; top: SMM derivatized with FDNB, middle: methylation of methionine with BurB, bottom: heat‐inactivated BurB. B) Mass spectra of native bis‐malleicyprol (top) and of ^13^C‐enriched bis‐malleicyprol (bottom) C) Synthesis of ^13^C‐labeled DMSP and subsequent complementation of *B. thailandensis PburΔburI* leads to incorporation of the C_3_ unit into **2**.

Having established the key steps to DMSP in *B. thailandensis* and with suitable null producers at hand, we performed chemical complementation. Therefore, we synthesized ^13^C_3_‐labeled DMSP from ^13^C_3_ acrylic acid (**10**, Figure 3 C). Supplementation of the *Pbur*Δ*burI* mutant with ^13^C_3_‐DMSP not only restored production of the malleicyprols (see Figure S4) but also enriched ^13^C in their cyclopropanol residue (Figure 3 B). These results unequivocally confirm DMSP as a key intermediate in the formation of the malleicyprol warhead.

Since DMSP accumulates in the Δ*burA* mutant, we reasoned that this unusual zwitterionic substrate would be activated and loaded onto the *bur* assembly line. In NRPS, adenylation (A) domains select, activate and load amino acids onto the assembly line and are thus regarded as gatekeepers.^18^ As bioinformatic substrate predictors^19^ failed on BurA‐A we generated a homology model of the A domain. In this way we noted the absence of the conserved aspartate (D235 in GrsA‐A) present in all α‐amino acid activating A domains.^18^ Consequently, BurA‐A was expected to select a non‐canonical starter unit lacking the α‐amino group usually bound by this aspartate. According to homology modeling, BurA‐A shares important binding pocket features with the A‐domain ATRR‐A activating glycine betaine.^20^ In both ATRR‐A and BurA‐A, the loop carrying conserved D235 (GrsA‐A) has been replaced with a shorter loop carrying hydrophobic residues (Figure 4 A; Figure S5). In another position, both binding pockets have an acidic residue (D606 in BurA‐A) well placed to interact with a positively charged substrate moiety such as the sulfonium group of DMSP.

**Figure 4:**
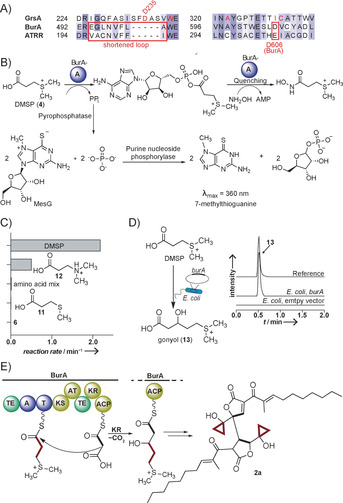
A) Sequence alignment of BurA‐A with the canonical A domain GrsA and the glycine betaine accepting A domain ATRR. B) Concept of the MesG/hydroxylamine A domain assay C) Specificity profile of BurA‐A; amino acid mix: all 20 proteinogenic amino acids (Figure S6). D) Heterologous production of gonyol in *E. coli*; UHPLC‐MS monitoring: EIC (*m*/*z* 179.0736) in positive ion mode; top, synthetic reference, middle, culture extracts of *E. coli Rosetta2* (DE3) expressing *burA* on plasmid pHIS8; bottom, *E. coli Rosetta2* (DE3) with empty pHIS8. E) Loading of DMSP onto BurA leads to production of gonyol and malleicyprols.

To verify whether BurA‐A selects and activates DMSP we cloned and expressed the gene fragment for the A domain (*burA‐A*) in *E. coli* BL21(DE3) and purified the His6‐tagged protein via Ni‐affinity. We probed the activity of BurA‐A with the MesG/hydroxylamine assay, which monitors pyrophosphate released during substrate adenylation in a coupled photometric assay (Figure 4 B).^21^ From a panel of carboxylic acids, BurA‐A shows highest activity for DMSP. In stark contrast, neither a mixture of the proteinogenic amino acids, **5** nor 3‐(methylthio)propionic acid (**11**) are activated (Figure 4 C). Apparently, the positively charged sulfonium group enables substrate binding. Replacing this group with protonated nitrogen in **12**, thus maintaining the positive charge, reduces activity more than four‐fold. In substrate saturation kinetics with DMSP, we determined a *k*_cat_ of 2.4 min^−1^ and a *K*_M_ of 0.15 mm (Figure S7). Altogether, these results indicate that DMSP is the preferred substrate of BurA‐A, making it the first adenylation domain that incorporates the osmolyte DMSP into a natural product assembly line.

An in silico analysis of the modular architecture of BurA suggested that DMSP, once loaded onto the thiolation (T) domain, would be elongated through Claisen condensation with malonyl‐ACP, and the resulting β‐keto intermediate transformed into the corresponding alcohol by the ketoreductase (KR) domain (Figure 4 E). To identify the downstream product of DMSP we heterologously reconstituted the assembly line. Therefore, we cloned and expressed *burA* in *E. coli*. Only when we employed IPTG for induction and supplemented DMSP, we detected production of compound **13** with *m*/*z* 179.0742 (positive ion mode) in XAD16 extracts (Figure 4 D). The presence of the same species in *B. thailandensis Pbur* (Figure 1 B) indicates that compound **13** is not an artifact generated in *E. coli* but actually formed in the intact *bur* pathway. By HR‐LCMS and MS^2^ comparison with an authentic reference we found that **13** is identical to the sulfonium acid gonyol.^22^ The structure of **13** agrees with our in silico prediction of the biosynthetic steps mediated by BurA. However, the discovery of this sulfonium intermediate of the malleicyprol assembly line is surprising, as **13** has been reported as a dominant zwitterion in the marine dinoflagellate *Gonyaulax polyedra*.^22^ Moreover, **13** is widely distributed as minor osmolyte in several phytoplankton groups.^23^ Previous studies have identified DMSP and acetate as the precursors of **13** in *G. polyedra*,^22^, ^24^ but the enzymes involved in gonyol biosynthesis have remained unknown. We now report BurA as the first enzyme involved in a gonyol biosynthetic pathway, and unexpectedly, it is a modular NRPS‐PKS hybrid. We reason that the sulfonium group represents a leaving group, likely as dimethylsulfide that enables cyclopropanol formation downstream of BurA. The enzymes and mechanisms involved in the cyclization are the subject of ongoing studies.

In summary, we have uncovered crucial steps in the biosynthetic pathway to the virulence factor malleicyprol employed by animal and human pathogenic *Burkholderia* species. Our findings have broad implications for ecology and synthetic biology. We describe BurB as a new *C‐S* bond‐forming enzyme in secondary metabolism that mediates a cryptic methylation to form the sulfonium group of the DMSP precursor. A role of this zwitterionic sulfonium acid in bacterial secondary metabolism is new. In contrast, DMSP is widely distributed in marine life, and metagenomics of known DMSP methyltransferase genes show that bacteria are significant producers of DMSP in marine environments.^25^ Our discovery of a new methyltransferase associated with DMSP biosynthesis and the identification of a PKS‐NRPS hybrid as a gonyol synthetase allows for genomics‐based identification of ecologically relevant producer strains. From a biosynthetic perspective DMSP is noteworthy as a novel PKS primer unit,^26^ and the sulfonium‐accepting adenylation domain BurA‐A is an important addition to the synthetic biology toolbox that opens up new possibilities for engineering polyketides and nonribosomal peptides.

## Conflict of interest

The authors declare no conflict of interest.

## Supporting information

As a service to our authors and readers, this journal provides supporting information supplied by the authors. Such materials are peer reviewed and may be re‐organized for online delivery, but are not copy‐edited or typeset. Technical support issues arising from supporting information (other than missing files) should be addressed to the authors.

Supplementary
