# Supplementary material for: Sulfonium Acids Loaded onto an Unusual Thiotemplate Assembly Line Construct the Cyclopropanol Warhead of a Burkholderia Virulence Factor
Source: Angew Chem Int Ed Engl. 2020 May 29;59(32):13511–5. doi: 10.1002/anie.202003958 (PMC7496086; doi:10.1002/anie.202003958)
Supplement: Supplementary file 1 — Supplementary [file ANIE-59-13511-s001.pdf]

Angewandte Chemie
Eine Zeitschrift der Gesellschaft Deutscher Chemiker

Supporting Information

Sulfonium Acids Loaded onto an Unusual Thiotemplate Assembly Line Construct the Cyclopropanol Warhead of a *Burkholderia* Virulence Factor

*Felix Trottmann, Keishi Ishida, Jakob Franke, Aleksa Stanišić, Mie Ishida-Ito, Hajo Kries, Georg Pohnert, and Christian Hertweck**

anie_202003958_sm_miscellaneous_information.pdf

Content

Bacterial Strains and General Culture Conditions 9
Suicide Plasmid Preparation for *B. thailandensis* mutants 9
Preparation of Gene Knockout Mutants of *B. thailandensis* E264 10
General Analytical Procedures 11
Heterologous Production and Purification of His$_6$-BurB 11
Methylation of L-Methionine Through BurB 12
Cloning and Purification of BurA-A 13
Adenylation Assay 14
Gonyol Synthesis 14
$^{13}C_3$ DMSP Synthesis 14
Stable Isotope Labeling of Malleicyprol with $^{13}C_3$-DMSP 14
Metabolite Extraction and Metabolomics Analysis 14
Construction of pHis$_8$-burA 15
Conversion of DMSP to Gonyol by Expression of *burA* in *E. coli* 15
Supplementary References 16

Supplementary Figures

Supplementary Figure 1. DMSP detection 2
Supplementary Figure 2. Extended Volcano Plot 3
Supplementary Figure 3. SMM detection 4
Supplementary Figure 4. Chemical complementation with 13C$_3$ DMSP 4
Supplementary Figure 5. Homology Model of BurA-A 5
Supplementary Figure 6. Specificity profile of BurA-A 6
Supplementary Figure 7. Michaelis Menten kinetics of BurA-A 6
Supplementary Figure 8. Confirmation of *B. thailandensis* mutants 11
Supplementary Figure 9. BurB purification. 12
Supplementary Figure 10. BurA-A purification. 13

Supplementary Tables

Supplementary Table 1. Primers used in this study 7
Supplementary Table 2. Vectors and plasmids used in this study 8
Supplementary Table 3. Bacterial strains used in this study 9

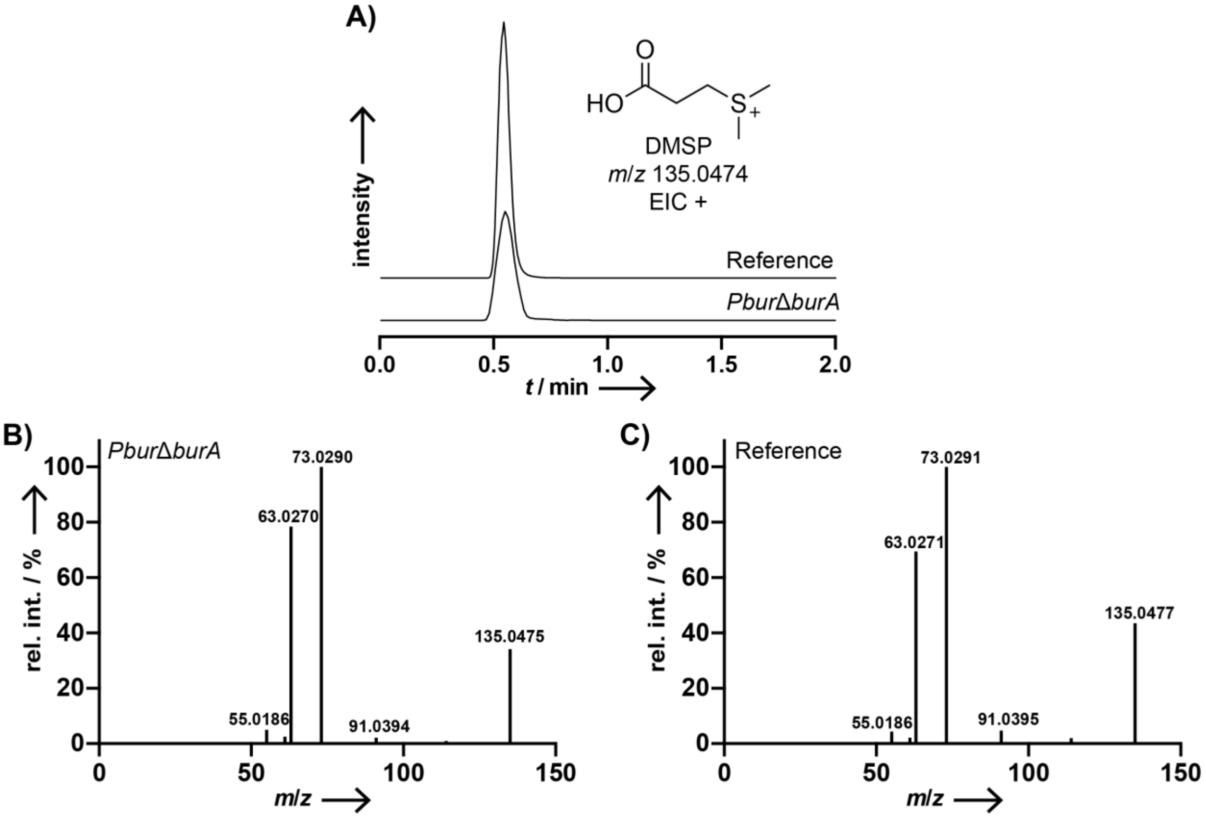

Supplementary Figure 1. DMSP detection in *B. thaialandensis PburΔburA* cell extracts. A) UHPLC-MS monitoring of DMSP (*m*/*z* 135.0474); top: commercial DMSP standard, bottom: *B. thailandensis PburΔburA* cell extract. Extracted Ion Chromatogram (135.0474 ± 5 ppm) in the positive ion mode; boxcar smoothing with five points as average applied. B) MS^2 spectrum of DMSP in *B. thaialandensis PburΔBurA*. C) MS^2 spectrum of commercial DMSP. A normalised collision energy of 55% was used for MS^2 experiments.

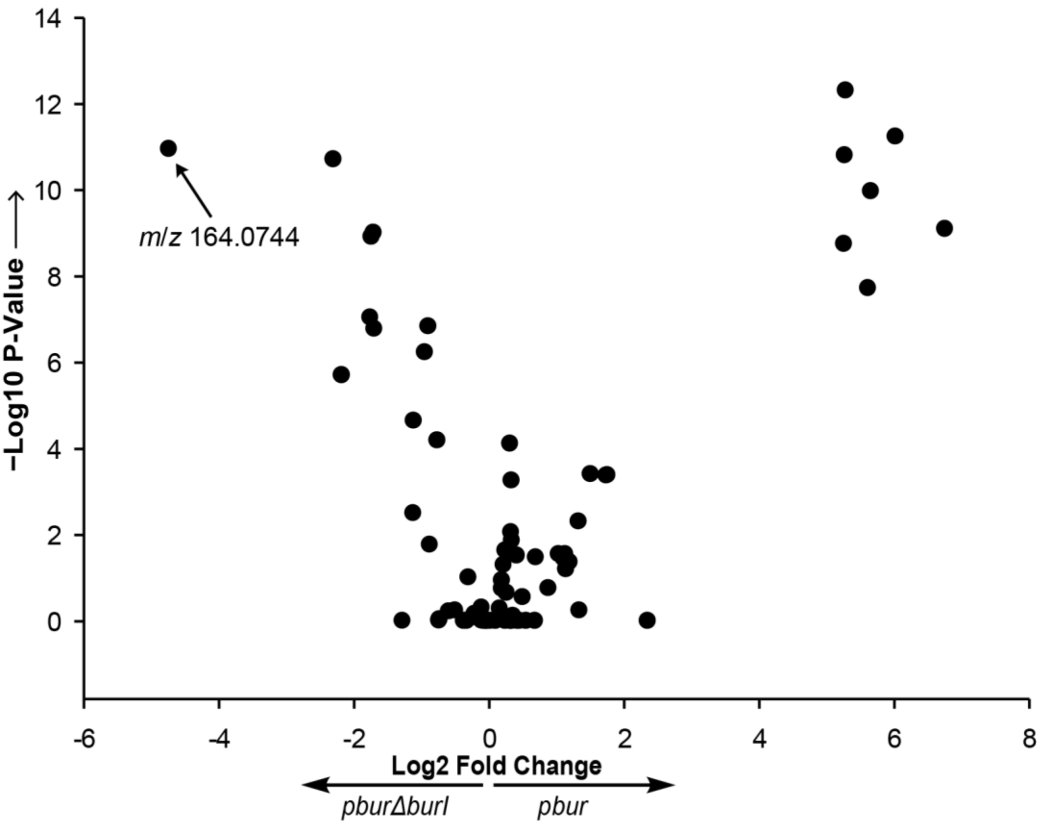

Supplementary Figure 2. Extended volcano plot from Figure 2B

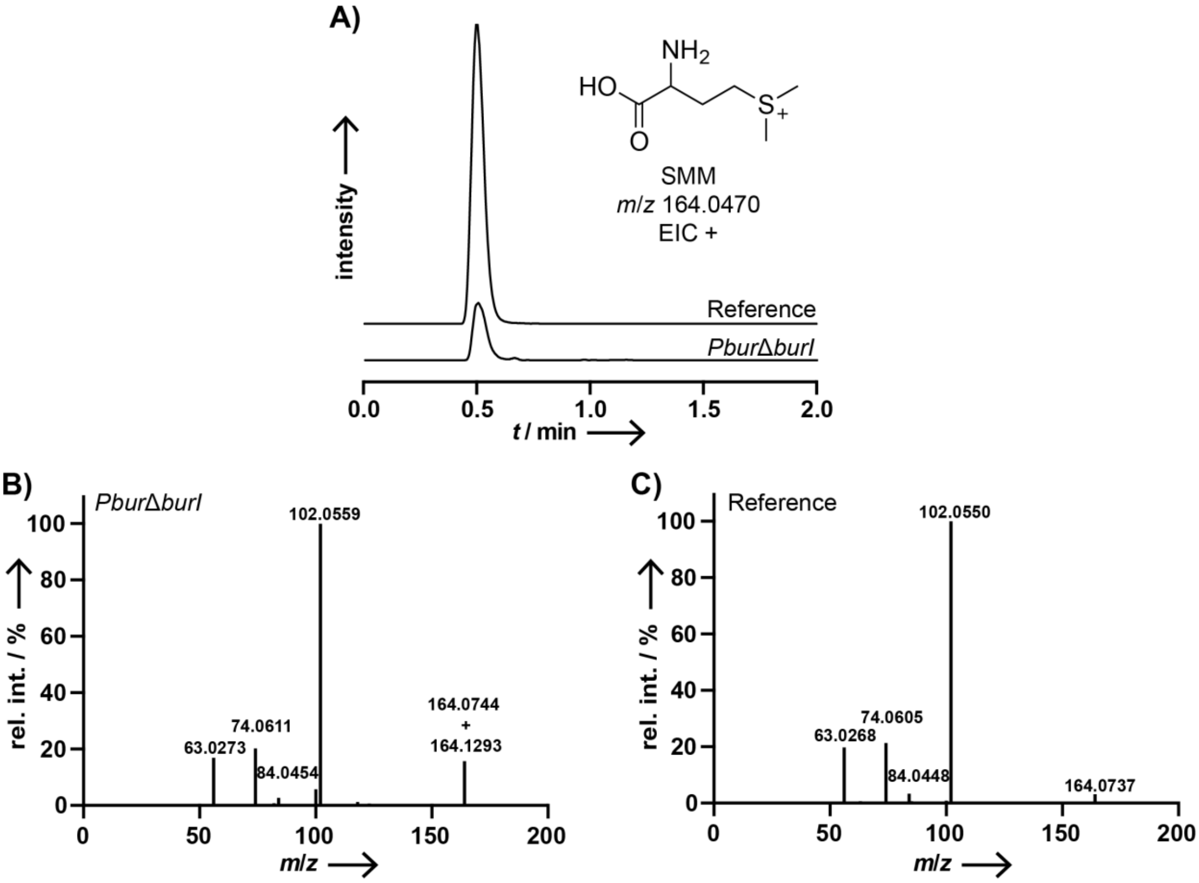

Supplementary Figure 3. SMM detection in *B. thailandensis PburΔBurI* cell extracts. A) UHPLC-MS monitoring of SMM (*m*/*z* 164.0470); top: commercial SMM standard, bottom: *B. thaialandensis PburΔBurI* XAD16N extract. Extracted Ion Chromatogram (164.0470 ± 5 ppm) in positive ion mode; boxcar smoothing with seven points as average applied. B) MS^2 spectrum of SMM in *B. thaialandensis PburΔBurI*. C) MS^2 spectrum of commercial SMM. A normalized collision energy of 10% was used for MS^2 experiments.

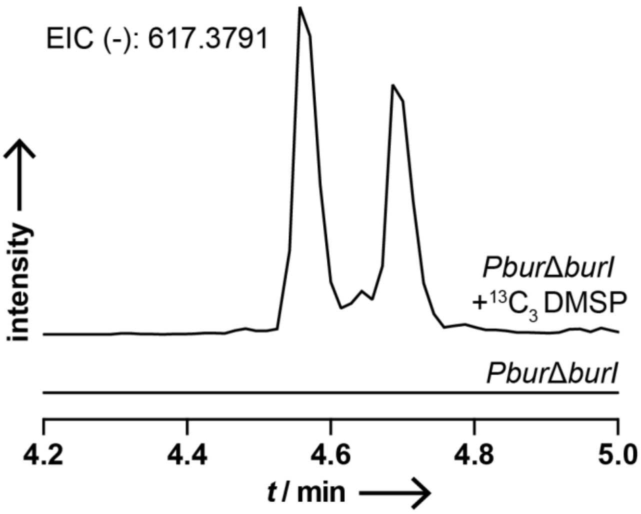

Supplementary Figure 4. UHPLC-MS monitoring of **c**hemical complementation of *B. thailandensis PburΔburI* with $^{13}C_3$ DMSP to restore malleicyprol production; top: complemented culture, bottom: *B. thailandensis PburΔburI* culture without complementation. Extracted Ion Chromatogram in the negative ion mode to show bis-malleicyprol with incorporation of six ^{13}C atoms (*m*/*z* 617.3791; $[M–H]^-$)

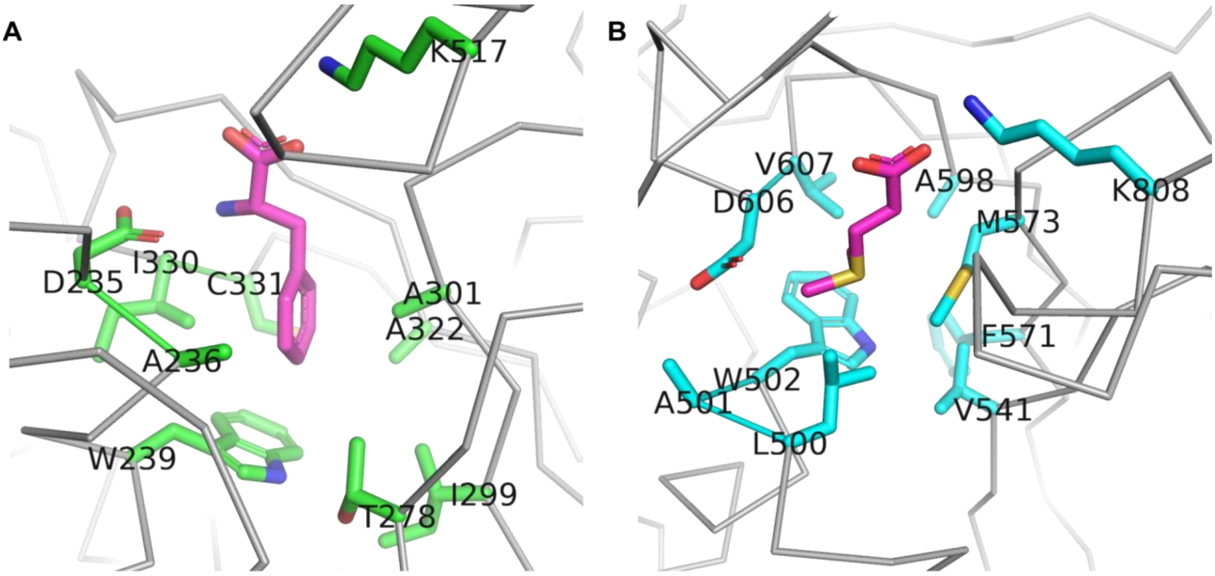

GrsA-A (PDB 1amu) / Phe

BurA-A model / DMSP

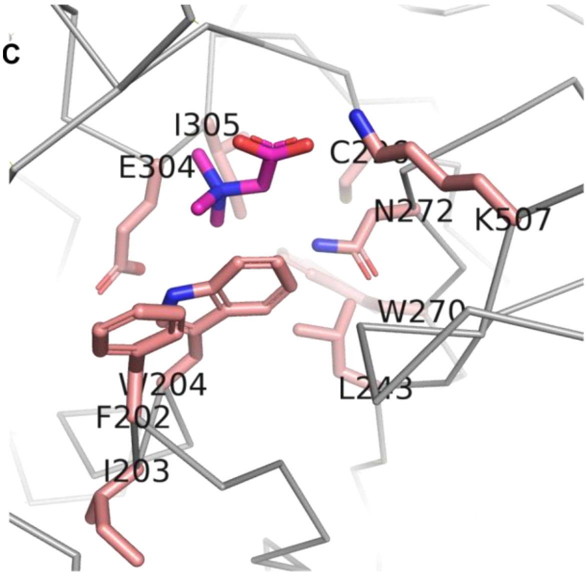

ATRR-A model / Gly-betaine

D

GrsA-A	BurA-A	ATRR-A
D235	L500	F202
A236	A501	I203
W239	W502	W204
T278	V541	L243
I299	F571	W270
A301	M573	N272
A322	A598	C296
I330	D606	E304
C331	V607	I305
K517	K808	K507

Supplementary Figure 5. Comparison of A-domain binding pockets. A) Crystal structure of the GrsA PheA-domain from gramicidin S biosynthesis (PDB code 1amu; [1]). The substrate is shown as sticks in magenta, the binding pocket residues in green. Residues K517 and D235 are highly conserved and bind the carboxylate and amino group of the substrate, respectively. B)-C) Homology models were prepared using the Swiss-Model server[2] with 1amu as a template. Sequence identities to the template are 25.3% (BurA-A) and 29.1% (ATRR-A[3]). Models of the substrates have been energy minimized in Chem3D (Perkin Elmer) and manually docked into the active sites in an orientation similar to that of Phe in GrsA-A. Hence, the positioning and conformation of the substrate is only an approximation. In both models, the loop bearing D235 in GrsA-A is replaced with a shorter loop not containing an acidic residue. Instead, an acidic residue (D606 in BurA-A and E304 in ATRR-A) is found in a position where it could electrostatically interact with the sulfonium or ammonium residue of the substrate, respectively. D) Binding pocket residues of GrsA-A, BurA-A and ATRR-A. It is not clear, whether the loop carrying the residues marked in blue has been modelled reliably.

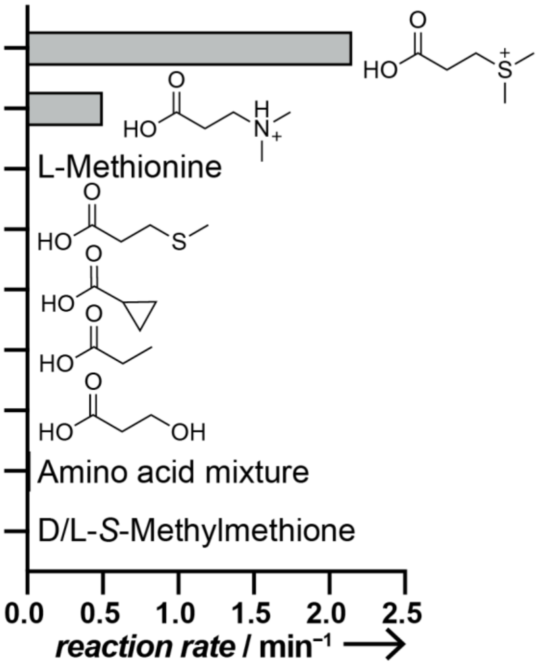

Supplementary Figure 6. Specificity profile of BurA-A, each substrate tested at a concentration of 2 mM in the MesG/hydroxylamine assay. Amino acid mixture constituted of all 20 proteinogenic amino acids each at a concentration of 1 mM. *S*-Methylmethione was used in its racemic form.

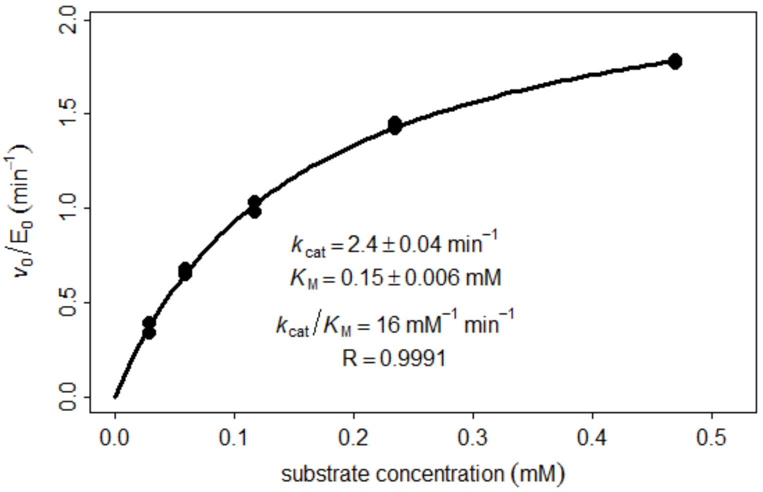

Supplementary Figure 7. Michaelis Menten kinetics of BurA-A with DMSP as substrate. Errors indicate the error of fit.

Supplementary Table 1. Primers used in this study.

Primer	Nucleotide sequence (5' to 3')	Source or reference
II2089-fw	CGA TGC AGG ACG GCG AAC GC	This study
II2089-PacI	GGT TTA ATT AAC GTA CAG GTG CGT GTG CGC G	This study
II2089-KpnI	GGT GGT ACC GAA CGT GCG CCT CGA CAT GG	This study
II2089-NheI	GGT GCT AGC TCG TGA TGC GCG GGT CTT CC	This study
burD-fw	TGA TGC GCG ACA AGG GCT AC	This study
burD-PacI	GGT TTA ATT AAC GAT CAG CGC GTC GAA TTC G	This study
burD-KpnI	GGT GGT ACC TTA TCT CGC CGC GAT GCG CG	This study
burD-NheI	GGT GCT AGC TCG TCC TGC ACG ACG AGA TG	This study
burE-KO1-HindIII	GGT AAG CTT TGA TCT CGG AGC GGC TCG TC	This study
burE-KO1-PacI	GGT TTA ATT AAC GAT CGA CAG CGG CGT CCA T	This study
burE-KO2-KpnI	GGT GGT ACC TTT CGC TGA CGG GCT CGG TC	This study
burE-KO2-NheI	GGT GCT AGC ACC GAA TCG AAG CCG AGC GC	This study
PLP-KO1-HindIII	GGT AAG CTT GAA GCC GAT CTG CTC GAC GC	This study
PLP-KO1-PacI	GGT TTA ATT AAG AAC TTC GTG ATG GTC GCG C	This study
PLP-KO2-KpnI	GGT GGT ACC CGT GAC GTG GCT GCA TTG CC	This study
PLP-KO2-NheI	GGT GCT AGC TGT AGC TGA GAT CGT CGG CG	This study
burB-fw-NheI	GGT GCT AGC ACG ATC ACC CTC ATC GAA CCT GTC	This study
burB-rv-HindIII	GGT AAG CTT TCA CGC GAG CGC GGC GGC CC	This study
JF007fw-BamHI	GGT GGT GGA TCC GAG CGC CCC GCG TCG ATC GT	This study
JF001rv-HindIII	GGT GGT AAG CTT TCA TCG CGC GTC GCT CGG CAC G	This study
2088fw-BamHI	GGA TCC AGG CAA ATA GTT TCA ACG AGC AAC G	This study
2088rv-PspOMI	GGG CCC GCC GAG ATC GAG CTG	This study
2088fw-PspOMI	GGG CCC GCG GTG TCG GTC GG	This study
2088rv-BspHI	TCA TGA AGT CAT GGC CGC CCG GG	This study
2088fw-BspHI	TCA TGA TGC GAT GCG AGG CC	This study
2088rv-HindIII	AAG CTT CTA CTC GTC CGC GAT GCC G	This study
MT2089fw	AAG AGG TCC GCC GGG TGC TC	This study
MT2089rv	AGG AGC GCC TGC CCG ATA TC	This study
burD-Fw2	CAC GGC AGC ACG TAT CTC GC	This study
burD-rv2	GAC CGG ATG GCC CGA ATA GG	This study
burE-fw1	GGG CGA ATA CGT CGA GGG CC	This study
burE-rv1	GTA GAT TCG CGT GGC CGC CG	This study
burI-fw	CGT GCT CGC CCG AGG AGT TG	This study
burI-rv	ACG TAT GCG ACG CGC AGC AC	This study

Supplementary Table 2. Vectors and plasmids used in this study.

Plasmid	Relevant characteristics	Source or reference
	**Vectors**	
pGEM T-easy	TA cloning vector; Amp^R	Promega
pJET 1.2	Blunt end cloning vector; Amp^R	Thermo Scientific
pGL42a	KO vector carrying *pheS* for Burkholderia species; Amp^R	[4]
pGL42aT251A	pGL42a carrying modified *pheS* (T251A); Amp^R	[5]
pET28a	*E. coli* expression vector with His_6-tag; Kan^R	Novagen
$pHis_8$-3-svp	*E. coli* expression vector with His_8-tag and *svp*; Kan^R	[6]
	**Plasmids**	
pGEM-II2089-KO1	pGEM T-easy containing a part of genes of *burA, B*; Amp^R	This study
pGEM-II2089-KO2	pGEM T-easy containing a part of genes of *burB, C*; Amp^R	This study
pGEM-Kan^R (fw)	Kan^R cassette with *Pac*I/*Kpn*I restriction sites; Amp^R, Kan^R	[7]
pGEM-Δ*burB*	Kan^R cassette was inserted into *burB*; Amp^R, Kan^R	This study
pGL42a-Δ*burB*	Δ*burB* fragment was exchanged a vector pGL42a; Amp^R, Kan^R	This study
pGEM-burD-KO1	pGEM T-easy containing a part of gene of *burC, D*; Amp^R	This study
pGEM-burD-KO2	pGEM T-easy containing a part of gene of *burD*; Amp^R	This study
pGEM-Δ*burD*	Kan^R cassette was inserted into *burD*; Amp^R, Kan^R	This study
pJET-burE-KO1	pGEM T-easy containing a part of gene of *burD, E*; Amp^R	This study
pJET-burE-KO2	pGEM T-easy containing a part of gene of *burE, F*; Amp^R	This study
pGL42aT251A-Δ*burE*	Kan^R cassette was inserted into *burE*; Amp^R, Kan^R	This study
pGEM-PLP-KO1	pGEM T-easy containing a part of gene of *burH, I*; Amp^R	This study
pGEM-PLP-KO2	pGEM T-easy containing a part of gene of *burI, J*; Amp^R	This study
pGL42a-Δ*burI*	Kan^R cassette was inserted into *burI*; Amp^R, Kan^R	This study
pJET-burB	pJET1.2 containing *burB* gene; Amp^R	This study
pET28a-burB	pET28a (+) containing *burB* gene; Kan^R	This study
pGEM-burA-A	pGEM T-easy containing adenylation domain gene in *burA*; Amp^R	This study
pET28a-burA-A	pET28a (+) containing adenylation domain gene in *burA*; Kan^R	This study
pET28a-burA	pET28a (+) containing *burA*; Kan^R	This study
$pHis_8$-burA	$pHis_8$-3-*svp* containing *burA*; Kan^R	This study

Bacterial Strains and General Culture Conditions

Burkholderia thailandensis wild-type strain E264 (DSM13276) was obtained from the DSMZ GmbH (Braunschweig). *B. thailandensis* E264 was cultured in MM9[8] liquid medium or LB agar at 30 °C. For mutant strains, tetracycline (45 µg mL^{-1}, *B. thailandensis Pbur*) and tetracycline (45 µg mL^{-1}) in addition to kanamycin (150 µg mL^{-1}, *B. thailandensis* double mutants) were used as selection markers.

Supplementary Table 3. Bacterial strains used in this study.

Strain	Relevant characteristics	Source or reference
	E. coli	
TOP10	General cloning host strain	Invitrogen
XL1-Blue	General cloning host strain; Tet^R	Stratagene
Rosetta2 (DE3)	Protein expression host strain carring rare codon; Cm^R	Novagen
BL21 (DE3)	Protein expression host strain	NEB
Rosetta2 (DE3) pET28a-*burB*	His_6-BurB producing strain; Cm^R, Kan^R	This study
Rosetta2 (DE3) pHis_8-*burA*	His_8-BurA producing strain; Cm^R, Kan^R	This study
Rosetta2 (DE3) pET28a-*burA-A*	His_6-BurA-A producing strain; Cm^R, Kan^R	This study
	B. thailandensis	
E264	Prototroph; environmental isolate	DSMZ
E264 *Pbur*	A promotor of *burA* was exchanged with Δ*PthaA*;Tet^R	[8]
E264 *Pbur*Δ*burA*	Kan^R cassette inserted into *burA*; Tet^R, Kan^R	[8]
E264 *Pbur*Δ*burB*	Kan^R cassette inserted into *burB*; Tet^R, Kan^R	This study
E264 *Pbur*Δ*burD*	Kan^R cassette inserted into *burD*; Tet^R, Kan^R	This study
E264 *Pbur*Δ*burE*	Kan^R cassette inserted into *burE*; Tet^R, Kan^R	This study
E264 *Pbur*Δ*burI*	Kan^R cassette inserted into *burI*; Tet^R, Kan^R	This study

Suicide Plasmid Preparation for *B. thailandensis* Mutants

Genomic DNA of *B. thailandensis* E264 was purified by using a Wizard® Genomic DNA Purification Kit (Promega). Two gene fragments containing *burB* were amplified by PCR with the primer pairs II2089-fw/II2089-PacI and II2089-KpnI/II2089-NheI using PCR extender Polymerase (5 PRIME), respectively. The amplicons were purified using the illustra GFX PCR DNA and Gel Band Purification Kit (GE Healthcare Life Sciences) and cloned into pGEM T-easy vector (Promega), resulting in pGEM-II2089-KO1 and pGEM-II2089-KO2, respectively. pGEM-Kan^R (fw) and pGEM-II2089-KO2 were restricted with *Pac*I/*Kpn*I and *Kpn*I/*Nhe*I, respectively, and then each *ca.* 1 kb gene fragment was purified with the above-mentioned kit. These gene fragments were ligated into *Pac*I/*Spe*I-restricted pGEM-II2089-KO1, generating pGEM-Δ*burB*.

Two gene fragments containing *burD* were amplified by PCR with the primer pairs burD-fw/burD-KO1-PacI and burD-KO2-KpnI/burD-KO2-NheI using Phusion polymerase (New England Biolabs)

followed by Taq Polymerase with dATP, and purified with the above-mentioned kit and subcloned into pGEM T-easy, to result in the vectors pGEM-*burD*-KO1 and pGEM-*burD*-KO2, respectively. pGEM-Kan^R (fw) and pGEM-*burD*-KO2 were restricted with *Pac*I/*Kpn*I and *Kpn*I/*Nhe*I, respectively. The obtained *ca.* 1 kb gene fragments were ligated into *Pac*I/*Spe*I-restricted pGEM-*burD*-KO1, generating pGEM-Δ*burD*. This plasmid was further restricted with *Psp*OMI/*Pst*I, and ligated into *Psp*OMI/*Pst*I-restricted pGL42a, to yield pGL42a-Δ*burD*.

Two gene fragments containing *burE* were amplified by PCR with the primer pairs burE-KO1-HindIII/burE-KO1-PacI and burE-KO2-KpnI/burE-KO2-NheI using the DeepVent polymerase (New England Biolabs), subsequently the amplicons were subcloned into pJET1.2, to yield pJET-*burE*-KO1 and pJET-*burE*-KO2, respectively. These plasmids and pGEM-Kan^R (fw) were restricted with *Hin*dIII/*Pac*I, *Kpn*I/*Nhe*I, and *Pac*I/*Kpn*I, respectively, and ligated into *Hin*dIII/*Spe*I-restricted pGL42aT251A, generating pGL42aT251A-Δ*burE*.

A gene fragment containing *burI* was amplified by Phusion polymerase followed by Taq Polymerase with dATP(New England Biolabs), using the primer pair PLP-KO1-HindIII/PLP-KO1-PacI. Another gene fragment containing *burI* was amplified by PCR extender polymerase with the primer pair PLP-KO2-KpnI/PLP-KO2-NheI. The obtained two gene fragments were purified with the above-mentioned kit and then subcloned into pGEM T-easy, to yield pGEM-PLP-KO1 and pGEM-PLP-KO2. These plasmids and pGEM-Kan^R (fw) were restricted with *Hind*III/*Pac*I, *Kpn*I/*Nhe*I, and *Pac*I/*Kpn*I, respectively, and ligated into *Hin*dIII/*Spe*I-restricted pGL42a, generating pGL42a-Δ*burI*.

Preparation of Gene Knockout Mutants of *B. thailandensis* E264

An overnight preculture (0.5 mL) of *B. thailandensis Pbur* in LB medium (2 mL) supplemented with tetracycline (45 µg mL^{-1}) was inoculated into LB medium (50 mL) supplemented with tetracycline (45 µg mL^{-1}) and cultured in a 300 mL buffled Erlenmeyer flask at 30 °C with orbital shaking until an OD_{600} from 0.5 to 0.8 was reached. Cultured cells were centrifuged at 2,500 × g and 20 °C for 10 min. The obtained cells were washed with a 300 mM sucrose solution (3 ×) and resuspended in 300 µL sucrose solution. Subsequently, 100 µL of the washed *B. thailandensis Pbur* cells were subjected to electroporation (200 kV) with knockout plasmids (2–5 µg, see above). The transformed cells were precultured in LB broth (1 mL) for 4–6 h at 30 °C with shaking and then plated on either LB or nutrient agar plates with tetracycline (45 µg mL^{-1}) and kanamycin (150 µg mL^{-1}). After 3 days, a few positive colonies were observed and confirmed by PCR (see below) using purified genomic DNA from the respective mutants.

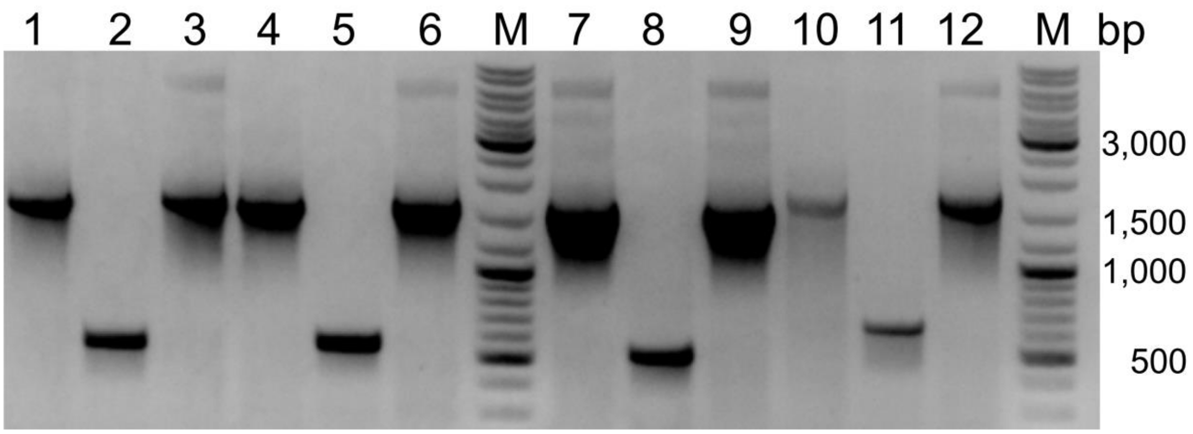

Supplementary Figure 8. Confirmation of *B. thailandensis* mutants by PCR using genomic DNA of *B. thailandensis Pbur*Δ*burB* (lane 1), *Pbur*Δ*burD* (lane4), *Pbur*Δ*burE* (lane 7), *Pbur*Δ*burI* (lane 10), wild type (lanes 2, 5, 8, 11; negative control), suicide plasmids (positive control), pGEM-Δ*burB* (lane 3), pGL42a-Δ*burD* (lane 6), pGL42aT251A-Δ*burE* (lane 9), pGL42a-Δ*burI* (lane 12). Primer pairs; MT2089-fw/rv (lanes 1–3), burD-fw2/rv2 (lanes 4–6), burE-fw1/rv1 (lanes 7–9), burI-fw/rv (lanes 10–12). Predicted size of amplicons; 1647 bp (lanes 1, 3), 569 bp (lane 2), 1602 bp (lanes 4, 6), 539 bp (lane 5), 1523 bp (lanes 7, 9), 492 bp (lane 8), 1713 bp (lanes 10, 12), 627 bp (lane 11).

General Analytical Procedures

LC-HRMS measurements were carried out on an UltiMate 3000 UHPLC (Thermo Fisher Scientific) coupled to a Thermo Fisher Scientific QExactive HF-X Hybrid Quadrupole-Orbitrap with an electrospray ion source using either a Kinetex 100-1.7 C_{18} column (50 × 2.1 mm, Phenomenex) and an elution gradient [solvent A: H_2O + 0.1% HCOOH, solvent B: CH_3CN, 5% to 100% B in 4.5 min, 100% B for 2 min, 100% B to 5% B in 0.001 min, 5% B for 1.5 min; flow rate: 0.7 mL min^{-1}, injection volume: 2 μL] or a Nucleodur 100-2 C_{18} column (100 × 2 mm, Macherey-Nagel) and an elution gradient [solvent A: H_2O + 0.1% HCOOH, solvent B: CH_3CN, 5% B for 0.5 min, from 5% to 100% B in 6.5 min, 100% B for 3 min, 100% B to 5% B in 0.01 min, 5% B for 2.9 min; flow rate: 0.4 mL min^{-1}, injection volume: 2 μL]. $HRMS^2$ measurements carried out on the same system operating in Parallel Reaction Monitoring (PRM) mode.

Heterologous Production and Purification of His_6-BurB

The gene fragment *burB* was amplified by PCR with the primer pair burB-fw-NheI/burB-rv-HindIII using the DeepVent polymerase and the resulting amplicon was purified by the illustra GFX PCR DNA and Gel Band Purification Kit followed by subcloning into pJET1.2, generating pJET-*burB*. This plasmid was restricted with *NheI*/*Hin*dIII and the obtained gene fragment *burB* was ligated into *Nhe*I/*Hin*dIII-restricted pET28a (+), to yield pET28a-*burB*. Subsequently the plasmid was introduced into *E. coli* Rosetta2 (DE3) for heterologous protein expression.

E. coli Rosetta2 (DE3) pET28a-*burB* was cultured in LB medium (2 mL) with chloramphenicol (25 μL mL^{-1}) and kanamycin (50 μL mL^{-1}) overnight. These cultured cells were inoculated into LB medium (50 mL) with added chloramphenicol (25 μL mL^{-1}) and kanamycin (50 μL mL^{-1}) in a 300 mL baffled Erlenmeyer flask and grown at 30 °C with orbital shaking until an OD_{600} = 1.5 was reached. The

bacterial culture was cooled in an ice bath for 20 min and IPTG (200 µM, final concentration) was added followed by further cultivation at 20 °C with orbital shaking for 18 h.
The overnight cultured cells were harvested by centrifugation at 20 °C and 8,000 × g for 5 min and kept at –25 °C until usage. 15 mL lysis buffer [50 mM Tris HCl (pH 8.0), 200 mM NaCl, 2 mg mL^{-1} lysozyme] were added to the cells and the mixture was incubated at 37 °C for 1 h. After DNase A (5 µL) was added, the cells were lysed by usage of a sonicator (BANDELIN SONOPULS HD2200) and centrifuged at 10,000 × g and 4 °C for 30 min. The resulting supernatant was filtered through a Chromafil® PET-45/15 MS (Macherey-Nagel) filter and subjected to a Ni-IDA agarose (Biontex) column (2 mL). The column was washed with 10 mL of 50 mM Tris HCl (pH 8.0), 200 mM NaCl, and 10 mM imidazole followed by elution with 10 mL of 50 mM Tris HCl (pH 8.0), 200 mM NaCl, and 200 mM imidazole, and 10 mL of 50 mM Tris HCl (pH 8.0), 200 mM NaCl, and 500 mM imidazole. Fractions containing His_6-BurB were collected and diluted 5 times with 50 mM Tris HCl (pH 8.0), 200 mM NaCl and subsequently concentrated on a Amicon Ultra-10K (MERCK-Millipore) filter to yield His_6-BurB (3.5 mg).

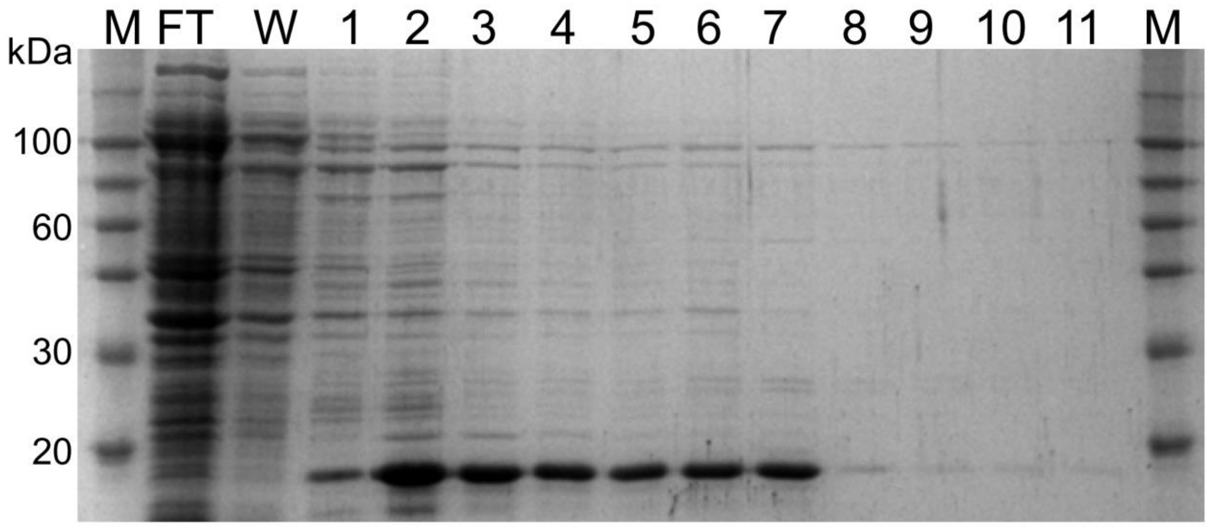

Supplementary Figure 9. 12% SDS-PAGE for His_6-BurB purification. M; marker, FT; flow through, W; wash, lanes 1–6; eluted fractions (200 mM imidazole), lanes 7–11; eluted fractions (500 mM imidazole). His_6-BurB has a size of ca. 21 kDa.

Methylation of L-Methionine Through BurB

Purified His_6-BurB (10 µM) was added to phosphate buffer (50 mM, pH 8.0) containing 20 mM NaCl, 1 mM L-methionine and 1 mM *S*-adenosylmethionine. As a control, purified BurB was heat-inactivated at 80 °C for 25 min and used in the same way. The resulting mixtures were incubated at 30 °C for 90 min. Subsequently, 50 µL of the reaction was diluted with 50 µL of a 0.5 M $NaHCO_3$ solution and 10 µL of a 1-fluoro-2,4-dinitrobenzene (DNFB) solution (1% w/v in acetonitrile) were added. After incubation at 60 °C for 60 min the reaction mixture was quenched with 12.5 µL HCl (2 M) and diluted with 122.5 µL methanol. As a reference, reaction buffer containing 1 mM *S*-methylmethionine was treated in the same way. The resulting solutions were filtered through a PTFE syringe filter and subjected to HR-LCMS analysis.

Cloning and Purification of BurA-A

A gene fragment coding for the adenylation domain of *burA* was amplified by PCR from genomic DNA of *B. thailandensis* E264 with the following primer pair: JF007fw-*Bam*HI and JF001rv-*Hin*dIII. The resulting PCR product was subcloned into the pGEM-T easy vector and verified by sequencing. The obtained subclone was excised and ligated into the *Bam*HI/*Hin*dIII sites of pET28a(+) to yield the overexpression plasmid pET28a-burA-A. The obtained plasmid was introduced into *E. coli* BL21 (DE3) for protein production and purification by transformation.

For protein purification, an overnight culture was inoculated at 1% (v/v) into LB medium (5 g L^{-1} yeast extract, 10 g L^{-1} tryptone, 5 g L^{-1} sodium chloride,) containing 50 µg mL^{-1} kanamycin and cultivated at 37 °C until an OD_{600} of 0.8 was reached. After cooling to 16 °C, 1 mM isopropyl 1-thio-β-D-galactopyranoside (IPTG) was added and the resulting culture incubated at 16 °C for 20 h. Subsequently, the cells were pelleted by centrifugation (6,000 × *g*, 4 °C, 10 min). The cell pellet was resuspended with lysis buffer (50 mM Tris-HCl, 1 M NaCl, 0.2% Trition X-100, 10 mM imidazole, pH 8.0) supplemented with 1 mg mL^{-1} lysozyme and 100 µM phenylmethylsulfonyl fluoride (PMSF) and incubated for 30 min at 37 °C. After cell disruption by using a homogeniser (BANDELIN SONOPULS GM2200; Germany) and removal of cell debris by centrifugation (6,000 × *g*, 4 °C, 30 min), the supernatant was loaded onto Ni Sepharose High Performance histidine-tagged protein purification resin (GE Healthcare Life Sciences) in a gravity flow column. After washing with wash buffer (50 mM Tris-HCl, 500 mM NaCl, 50 mM imidazole, 10% glycerol, pH 8.0) the protein was eluted with elution buffer (50 mM Tris-HCl, 500 mM NaCl, 500 mM imidazole, 10% glycerol, pH 8.0) and subsequently used in the adenylation assay. Protein concentration was determined using tryptophan absorbance at 280 nm and a calculated extinction coefficient (60,390 M^{-1} cm^{-1}). Absorbance was determined with a 2.5 µL sample in a Take3 plate on an Epoch2 microplate reader (BioTek).

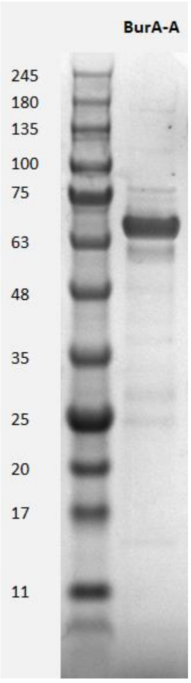

Supplementary Figure 10. SDS-PAGE of BurA-A. A Bolt 4-12% Bis-Tris Plus Gel (ThermoFisher Scientific) was run with MES-SDS buffer and stained with Quick Coomassie stain (Serva).

Adenylation Assay

The adenylation activity of BurA-A was measured with a range of substrates by using the MesG/hydroxylamine assay which was performed as described previously with minor modifications.[9] Reactions contained 50 mM TRIS (pH 7.6), 5 mM $MgCl_2$, 100 µM 7-methylthioguanosine (MesG), 150 mM hydroxylamine (adjusted to pH 7.5-8 with NaOH), 5 mM ATP (A2383, Sigma), 1 mM TCEP, 0.4 U/mL inorganic pyrophosphatase (I1643, Sigma), 1 U mL^{-1} purine nucleoside phosphorylase from microorganisms (N8264,Sigma), 5 µM BurA-A and 2 mM substrates. In flat-bottom 384-well plates (781620, Brand) reactions were started in 100 µL volume by addition of enzyme and the absorbance was followed at 355 nm on a Synergy H1 (BioTek) microplate reader at 30 °C. Background activity was recorded in wells containing buffer without substrate and the obtained slopes were subsequently subtracted. Each substrate concentration was measured in duplicates. Initial velocities were divided by the slope of a pyrophosphate calibration curve to obtain the pyrophosphate release rate. For determining Michaelis Menten parameters for DMSP, initial velocities were recorded in a range from 0.0018 to 0.47 mM DMSP at 1 µM BurA-A. Initial velocities divided by the enzyme concentration (v_0/[E_0]) were plotted against the substrate concentration and fit to the Michaelis-Menten equation by nonlinear regression using R version 3.4.2.

Gonyol synthesis

Gonyol was prepared by following a known procedure[10] from ethyl-bromoacetate and 3-(methylthio)-propionaldehyde through reformatsky reaction followed by ester hydrolysis and subsequent methylation with iodomethane.

$^{13}C_3$ DMSP synthesis

$^{13}C_3$ DMSP was synthesised according to Chambers *et. al.*[11] as hydrochloride from $^{13}C_3$ acrylic acid and dimethylsulfide by bubbling gaseous HCl into a solution of both educts in dichloromethane for 20 minutes and subsequent concentration *in vacuo*.

Stable isotope labelling of Malleicyprol with $^{13}C_3$-DMSP

*B. thailandensis Pbur*Δ*burI* was grown in a 300 mL baffled Erlenmeyer flask filled with 100 mL MM9 medium supplemented with 45 mg L^{-1} tetracycline, 150 mg L^{-1} kanamycin and 137 mg L^{-1} $^{13}C_3$ DMSP at 30 °C with shaking at 150 rpm for 24 h. Subsequently, the culture was extracted with ethyl acetate (2 ×), concentrated *in vacuo* and redissolved in methanol for LC-HRMS analysis.

Metabolite extraction and metabolomics analysis

B. thailandensis E264, *B. thailandensis Pbur*, *B. thailandensis Pbur*Δ*burA*[8], *B. thailandensis Pbur*Δ*burB*, *B. thailandensis Pbur*Δ*burI*, *B. thailandensis Pbur*Δ*burD* and *B. thailandensis Pbur*Δ*burE* were each grown in a 300 mL baffled Erlenmeyer flask filled with 100 mL MM9 medium

supplemented with the appropriate antibiotic and 2% (w/v) XAD16N at 30 °C with shaking at 150 rpm for 24 h. Subsequently, the XAD16N resin was separated from the culture broth by filtration through Miracloth (Merck Millipore). The XAD16N resin was washed with water and eluted with methanol (40 mL) followed by elution with ethyl acetate (20 mL). Both eluted fractions were combined and concentrated under reduced pressure to yield supernatant extracts. For cell extracts, 50 mL of filtered culture broth was pelleted by centrifugation (6000 g, 10 min). Subsequently the pellet was resuspended in 20 mL methanol, sonicated and incubated for 1 h. Cell debris was removed by centrifugation (6000 g, 10 min) and filtration through a 0.2 µm PTFE ROTILABO syringe filter. The resulting methanol solution was concentrated *in vacuo* to yield cell extracts. Cell and supernatant extracts were dissolved in methanol to yield a concentration of 1.75 mg mL^{-1} of crude extract mass for supernatant and 1.64 mg mL^{-1} for cell extracts. All extracts were subjected to LC-HRMS analysis in two technical replicates for subsequent metabolomics analysis using the software Compound Discoverer (2.1, SP1) from Thermo Fisher Scientific. Additionally MM9 medium substituted with tetracycline (45 µg mL^{-1}) and kanamycin (150 µg mL^{-1}) was treated in the same way as mentioned above for supernatant extracts. This medium control was used to subtract medium components when supernatant extracts were analysed. Both extract types were analysed using a Metabolomics Workflow from Compound Discoverer with a Pattern Scoring node to identify sulphur-containing metabolites. All genotypes were compared to each other using differential analysis.

Construction of pHis$_8$-burA

Three gene fragments II2088-1, II2088-2, and II2088-3 in *burA* were amplified by PCR with the primer pairs 2088-fw-BamHI/2088-rv-PspOMI, 2088-fw-PspOMI/2088-rv-BspHI, and 2088-fw-BspHI/2088-rv-HindIII using PCR extender polymerase, respectively. The resulting three amplicons were purified by the illustra GFX PCR DNA and Gel Band Purification Kit and subcloned into pGEM T-easy, to obtain pGEM-II2088-1, pGEM-II2088-2, and pGEM-II2088-3. These three plasmids were restricted with *Bam*HI/*Psp*OMI, *Psp*OMI/*Bsp*HI, and *Bsp*HI/*Hin*dIII, respectively, and gene fragments for *burA* were ligated into *Bam*HI/*Hin*dIII-restricted pET28a (+), generating pET28a-burA. This plasmid was restricted with *Bam*HI/*Hin*dIII and the resulting burA was ligated into *Bam*HI/*Hin*dIII-restricted pHis$_8$-3-svp, to obtain pHis$_8$-*burA*.

Conversion of DMSP to Gonyol by Expression of *burA* in *E. coli*

A 300 mL baffled Erlenmeyer flask containing 75 mL MM9[8] liquid medium supplemented with 25 µg mL^{-1} chloramphenicol and 50 µg mL^{-1} kanamycin was inoculated with 1% of an overnight culture of *E. coli* Rosetta2 (DE3) expressing His$_8$-BurA. *E. coli* Rosetta2 (DE3) containing an empty vector (pHis$_8$-3-svp) was used as negative control. The cultures were grown at 37 °C with shaking at 150 rpm until an OD_{600} of 1.3 was reached. After cooling to 16 °C, the cultures were supplemented with 5.1 mg DMSP, heterologous protein production was induced with 0.5 mM IPTG and the resulting cultures were incubated at 16 °C with shaking at 150 rpm for 18 h. Subsequently, 1.3 g XAD16N resin was added per culture followed by incubation with shaking at 100 rpm for 30 min. The XAD16N resin was harvested from the culture broth by filtration through Miracloth (Merck Millipore), washed with water and eluted with methanol (40 mL) followed by elution with ethyl acetate (20 mL). Both eluted fractions were combined, concentrated under reduced pressure, redissolved in methanol to a

concentration of 2.33 mg mL^{-1} of crude extract mass, filtered through a PTFE syringe filter and subjected to HR-LCMS analysis.

Supplementary References

[1] E. Conti, T. Stachelhaus, M. A. Marahiel, P. Brick, *EMBO J.* **1997**, *16*, 4174-4183.
[2] M. Biasini, S. Bienert, A. Waterhouse, K. Arnold, G. Studer, T. Schmidt, F. Kiefer, T. G. Cassarino, M. Bertoni, L. Bordoli, T. Schwede, *Nucleic Acids Res.* **2014**, *42*, W252-W258.
[3] Y. Hai, A. M. Huang, Y. Tang, *Proc. Natl. Acad. Sci. U.S.A.* **2019**, *116*, 10348-10353.
[4] G. Lackner, N. Moebius, C. Hertweck, *ISME J.* **2011**, *5*, 252-261.
[5] R. Hermenau, J. L. Mehl, K. Ishida, B. Dose, S. J. Pidot, T. P. Stinear, C. Hertweck, *Angew. Chem. Int. Ed.* **2019**, *58*, 13024-13029.
[6] a) J. M. Jez, J.-L. Ferrer, M. E. Bowman, R. A. Dixon, J. P. Noel, *Biochemistry* **2000**, *39*, 890-902; b) C. Sánchez, L. Du, D. J. Edwards, M. D. Toney, B. Shen, *Chem. Biol.* **2001**, *8*, 725-738.
[7] F. Trottmann, J. Franke, K. Ishida, M. García-Altares, C. Hertweck, *Angew. Chem. Int. Ed.* **2019**, *58*, 200-204.
[8] J. Franke, K. Ishida, C. Hertweck, *Angew. Chem. Int. Ed.* **2012**, *51*, 11611-11615.
[9] B. P. Duckworth, D. J. Wilson, C. C. Aldrich, *Methods Mol. Biol.* **2016**, *1401*, 53-61.
[10] B. Gebser, G. Pohnert, *Mar. Drugs* **2013**, *11*, 2168-2182.
[11] S. T. Chambers, C. M. Kunin, D. Miller, A. Hamada, *J. Bacteriol.* **1987**, *169*, 4845-4847.
